# Exosomal miR-152-5p and miR-3681-5p function as potential biomarkers for ST-segment elevation myocardial infarction

**DOI:** 10.1016/j.clinsp.2022.100038

**Published:** 2022-06-22

**Authors:** Xiaozhu Chen, Fengrong Huang, Yunhong Liu, Shujun Liu, Gangwen Tan

**Affiliations:** a Department of Ultrasound, People's Hospital of Longhua Shenzhen, Guangdong, China; b Department of Cardiology, People's Hospital of Longhua Shenzhen, Guangdong, China; c Clinical Laboratory, People's Hospital of Longhua Shenzhen, Guangdong, China

**Keywords:** microRNAs, Real-time three-dimensional spot tracking echocardiography (RT3D-STE), ST-segment elevation myocardial infarction (STEMI), Non-ST-segment elevation myocardial infarction (NSTEMI), Exosomes, Acute myocardial infarction

## Abstract

**Background:**

The strain parameters of Real-Time Three-Dimensional Spot Tracking Echocardiography (RT3D-STE) are GLS, GAS, GRS, and GCS, while each index can significantly diagnose Acute Myocardial Infarction (AMI) patients, but none of them can distinguish between NSTEMI and STEMI. MicroRNAs (miRNAs) play essential roles in Acute Myocardial Infarction (AMI), but little is known about the value of exosome miRNA combined with Real-Time Three-Dimensional Spot Tracking Echocardiography (RT3D-STE) between ST-segment Elevation Myocardial Infarction (STEMI) and Non-ST-segment Elevation Myocardial Infarction (NSTEMI).

**Aim:**

To estimate the exosomal miRNAs related to strain parameters of RT3D-STE as biomarkers for early detection of STEMI and NSTEMI.

**Methods:**

The present study collected plasma samples from thirty-four (34) patients with AMI (including STEMI and NSTEMI) and employed high-throughput sequence technology and real-time quantitative polymerase chain reaction (RT-qPCR) to identify the differentially expressed miRNAs. The Pearson correlation coefficient is used to measure the strength of a linear association between differentially expressed miRNAs and strain parameters of RT3D-STE.

**Results:**

Twenty-eight (28) differentially expressed exosomal miRNAs were universally identified between STEMI, NSTEM, and normal groups. Among them, there are 10 miRNAs (miR-152-5p, miR-3681-5p, miR-193a-5p, miR-193b-5p miR-345-5p, miR-125a-5p, miR-365a-3p, miR-4520-2-3p, hsa-miR-193b-3p and hsa-miR-5579-5p) with a Pearson correlation greater than 0.6 with RT3D-STE strain parameters. Especially, miR-152-5p and miR-3681-5p showed the most significant correlation with RT3D-STE strain parameters. Target genes of these 10 miRNAs are analyzed for Gene Ontology (GO) and Kyoto Encyclopedia of Genes and Genomes (KEGG) pathways enrichment, and they were found to be mainly involved in the cellular metabolism processes and HIF-1 signaling pathway. RT-qPCR verified the significant differential expression of miR-152-5p and miR-3681-5p between STEMI and NSTEM groups.

**Conclusion:**

RT3D-STE and exosome miRNAs can be used as a hierarchical diagnostic system in AMI. If the RT3D-STE is abnormal, the exosome miRNAs can be detected again to obtain more detailed and accurate diagnostic results between STEMI and NSTEM groups. Exosomal miR-152-5p and miR-3681-5p may serve as potential biomarkers for ST-segment elevation myocardial infarction.

## Highlights

• RT3D-STE and exosome miRNAs can be used as a hierarchical diagnostic system in AMI.
• Exosomal miR-152-5p and miR-3681-5p function as potential biomarkers for ST-segment elevation myocardial infarction.

## Introduction

Acute Myocardial Infarction (AMI) is one of the most serious clinical types of coronary heart disease. After complete occlusion of the blood vessels, due to lack of blood flow perfusion in the myocardium, the corresponding myocardial necrosis may result in severe heart failure, fatal arrhythmia, cardiogenic shock, or even cardiac arrest, which seriously threatens the life of patients.^1^ Early detection is crucial in improving clinical outcomes and decreasing mortality. AMI can be divided into ST-segment Elevation Myocardial Infarction (STEMI) and Non-ST-segment Elevation Myocardial Infarction (NSTEMI). STEMI usually occurs in patients on the basis of coronary atherosclerosis, which is a rich source of red blood cells and red fibrin thrombus and leads to complete suspension of coronary blood flow.^2^ NSTEMI usually occurs when an unstable plaque forms as a white, rich platelet thrombus, inducing an incomplete occlusion in the coronary artery, but a small amount of blood can still be present.^3^^,^^4^ Although the Electrocardiogram (ECG) signs of ST-segment elevation are sensitive and specific signs of Total coronary Occlusion (TO) in STEMI patients, only about 25.5%‒34% of NSTEMI patients are found to have TO. Patients suffering from TO are commonly underdiagnosed, receive delayed intervention, and have increased rates of complications and mortality. Therefore, finding a method that can diagnose STEMI and NSTEMI shortly after the onset of symptoms has important clinical significance. It may reduce mortality and improve patient's prognosis.^3^^,^^5^

Circulating biomarkers play an important role in the diagnosis of cardiovascular diseases. For example, Cardiac Troponins T (cTNT) and creatine kinase MB act as sensitive and specific tests for myocardial damage, yet, they may be negative early in the process of ischemia.^6^ MicroRNAs (miRNAs) are single-stranded RNA molecules of approximately 21‒23 nucleotides in length. These have been identified and investigated for their possible diagnostic and prognostic utility in cardiovascular disease in recent years.^6^ Exosomes are lipid bilayer vesicles with a diameter of 30‒150 nm secreted by cells, containing a large number of proteins, nucleic acids, lipids, and other bioactive substances. These are considered as key components of intercellular communication.^7^ Increasing evidence has indicated that exosomes can play an important role in the diagnosis of myocardial ischemia and infarction in patients with AMI.^8^ Studies have shown that the expressions of some exosome miRNAs, such as miR-1, miR-133, miR-208a, and miR-499 have significant differences in patients with AMI compared with the normal group.^9^ The difference in exosomal miRNAs expression profile in STEMI and NSTEMI has been studied deeply. Circulating cardiac-specific exosome microRNAs is a promising indicator for the early diagnosis of STEMI and NSTEMI. Therefore, it may be of great value to explore exosome miRNAs combined with echocardiography and other methods to assess the diagnosis of MI.

In order to reliably evaluate the changes in cardiac function in patients with myocardial infarction and the functional state of the ischemic myocardium, echocardiography can assess the changes in left ventricular function, shape, and size, including left ventricular volume enlargement, shape change, myocardial thinning in infarcted segments, and thickening in non-infarcted segments.^10^^,^^11^ In particular, Real-Time Three-Dimensional Spot Tracking Echocardiography (RT3D-STE) can track the myocardial spot movement in three-dimensional space and synchronously measure the Globled Longitude Strain (GLS), Globled Radial Strain (GRS), Globled Circumferied Strain (GCS) and Globled Area Strain (GAS) of the left ventricle, in order to show the strain changes of the ischemic myocardium more sensitively.^12^ RT3D-STE displays the strain values of the 17 myocardial segments of the left ventricle during the same cardiac cycle, which can quantitatively evaluate the infarct range of AMI, the overall left ventricular and local myocardial function, and repeated inspections without trauma.^13^ It may become an important means of early diagnosis of AMI and evaluation of risk stratification.

This study is aimed to analyze the correlation between exosomal miRNAs and strain parameters of RT3D-STE, possibly providing a valuable basis for the diagnosis and prognosis of early STEMI and NSTEMI.

## Materials and methods

### Sample collection

The present study was approved by the Research Ethics Committee of Shenzhen Longhua People's Hospital (n° KY20200801). All ethical procedures conformed to the principles of the 1964 Declaration of Helsinki and its latest 2008 amendments. The research was conducted with the informed consent of each participant, and all participants provided signed informed consent. Thirty-four (34) samples were collected from Shenzhen Longhua People's Hospital from December 2019 to May 2020.

### Exosome isolation and miRNA extraction

The clinical specimens were thawed on ice. Then, exosomes were isolated from the plasma using an exoQuick precipitation kit (System Biosciences, USA) which was used according to the manufacturer's protocol. The total RNA, including the miRNAs, was extracted using the RNA Isolation Reagent kit (Vazyme, Nanjing, China) and was used according to manufacturer's instructions. In addition, after adding isopropyl alcohol, 1 μg of glycogen was required for each sample. After this step, the mixture was stored at -80°C for overnight to precipitate small RNA. The purity and concentration were determined using Bioanalyzer 2100 (Agilent Technologies, Santa Clara, CA).

### Detection of exosomes

The pooled exosome extracts were diluted with PBS at a ratio of 1:50, gently blown and mixed. Then, 10 uL of the diluent was dropped on a 2 mm carbon-bearing copper network. It was allowed to stand in room temperature for five minutes, and the excess liquid was absorbed using filter paper. Then, negative staining was performed with 3% sodium phosphotungstate solution for five minutes, and it was dried at room temperature and photographed.

Nanoparticle tracking analysis (NTA, Particle Metrix, Germany) was used to identify the exosomes. The NTA software ZetaView 8.04.02 SP2 (Particle Metrix, Germany) was used for data acquisition and processing according to the manufacturer's instructions. The supernatant was filtered through a 0.45 μm PES filter, mixed with 0.5 M EDTA, pH 8.0 (Life Technologies, USA), and then adjusted to pH 4.2. The solution was centrifuged at 300g for 10 min at 4°C, the supernatant was collected, and the pH was adjusted to 7.0. The ambient temperature was set at 24°C, while background extraction and automatic settings were applied for the minimum expected particle size, minimum track length, and blur. The samples were diluted with the ratio of 1:50 following sterile filtration with vesicle-free DPBS. Each experiment was r performed in triplicate.

### The miRNA sequencing and differential expression analysis

The miRNA library was constructed using the QIAseq miRNA Library Kit (QIAGEN, Germany). The main experimental steps included 3′ ligation, 5′ ligation, reverse transcription, QIAseq miRNA NGS (QMN) bead preparation, cDNA cleanup, library amplification using HT plate indices, and library amplification using the tube indices. The library, after quality control, was sequenced by Illumina HiSeq 2500 with the SE50 strategy. The investigators employed The Cutadapt software to cut off the adapters from both ends of the raw reads while retaining the reads longer than 17 nucleotides. The FANse3 ultra-high-precision sequence alignment algorithm^14^^,^^15^ was used to align the reads obtained from each sample with the reference sequence of the species (Human mature miRNA, miRBase release 22.1). The mapping parameter is -E5% ‒ indel -S14. The transcripts per million reads (TPM) were calculated to normalize for the RNA depth of sequencing. miRNAs with reading counts greater than or equal to 10 were considered to be expressed, while read counts of less than 10 were considered to indicate no expression. For the gene sequencing data, differential expression analysis was performed using the edgeR package, which is a statistical method based on the negative binomial distribution. The miRNA was defined as differentially expressed when |logFC| was > 1 and FDR (false discovery rate) was < 0.01. miRNA with logFC > 1 was considered to be upregulated, while miRNAs with logFC < -1 were considered to be downregulated.

### Prediction of gene ontology (GO) terms and pathways by miRNAs targeted gene

Genes targeted by differentially expressed miRNAs that had a strong correlation with GAS, GLS, GCS and GRS were predicted with the use of a public miRTarBase database and categorized according to their Gene Ontology (GO) terms. This can provide comprehensive information on gene function using the topGO (version 2.18.0) software with Fisher's Exact test. Pathway identification was performed with the KOBAS 2.0 software and hypergeometric tests via the Kyoto Encyclopedia of Genes and Genomes (KEGG) database.

### Real-time quantitative polymerase chain reaction

To verify results, the screened miRNAs were analyzed by Real-Time quantitative Polymerase Chain Reaction (RT-qPCR). Total RNA was extracted by the TRIpure Total RNA Extraction Reagent method (ELK Biotechnology, China). RNA concentration and purity were determined by a NanoDrop 2000 (Thermo Fisher Scientific). Takara 638314 (Takara, Japan) was used to synthesize cDNA in one step. PCR amplifications were performed using a StepOnePlus System (Thermo Fisher Scientific) with an initial denaturation step at 10s at 95°C, followed by 40 cycles of 95°C for 5s and 60°C for 20s, then 60s at 95°C, 30s at 55°C, and 95°C for 30. The internal reference gene is U6. The primer sequences used for the evaluated genes are listed in Supporting Table 1.

**Table 1:** Comparison of three groups of clinical data.

Item	NC (n = 14)	STEMI (n = 10)	NSTEMI (n = 10)
Age (years)	50.05±4.80	53.35±10.45	52.85±6.75
CKMB(U/L)	16.00 (10.00, 24.00)	249.00 (129.95, 2000.00)^a^	176.50 (31.80, 400.00)^a^^,^^b^
cTnI (ng/mL)	0.02 (0.01, 0.03)	17.05 (1.47, 80.00)^a^	8.56(0.93, 80.00)^a^^,^^b^
CK(U/L)	32.50 (20.00, 156.00)	3232.00 (886.00,7620.00)^a^	690.00 (106.00, 1074.50)^a^^,^^b^
GAS	−22.06±4	−12.93±2.1^a^	−12.91±1.84^a^
GLS	−13.93±2.35	−7.71±0.56^a^	−7.45±0.81^a^
GCS	−14.06±2.05	−6.11±2.01^a^	−7.21±2.07^a^
GRS	29±3.46	16.66±2.57^a^	16.66±2.44^a^

a Compared with the normal group, p < 0.01.

b Compared with the STEMI group, p < 0.05

### Statistical analysis

Cycle threshold (Ct) values were processed by LightCycler 480 v1.5.0.39 software. The expression value of miRNAs relative to internal controls was calculated using the 2^−△△Ct^ method. Statistical analysis was performed with SPSS software. Data were tested for significance with the nonparametric Mann-Whitney *U* test. A p-value <0.05 was considered to be significant. Pearson correlation analysis was performed by SPSS 22.0.

## Results

### Comparison of clinical data

**Fig. 1:**
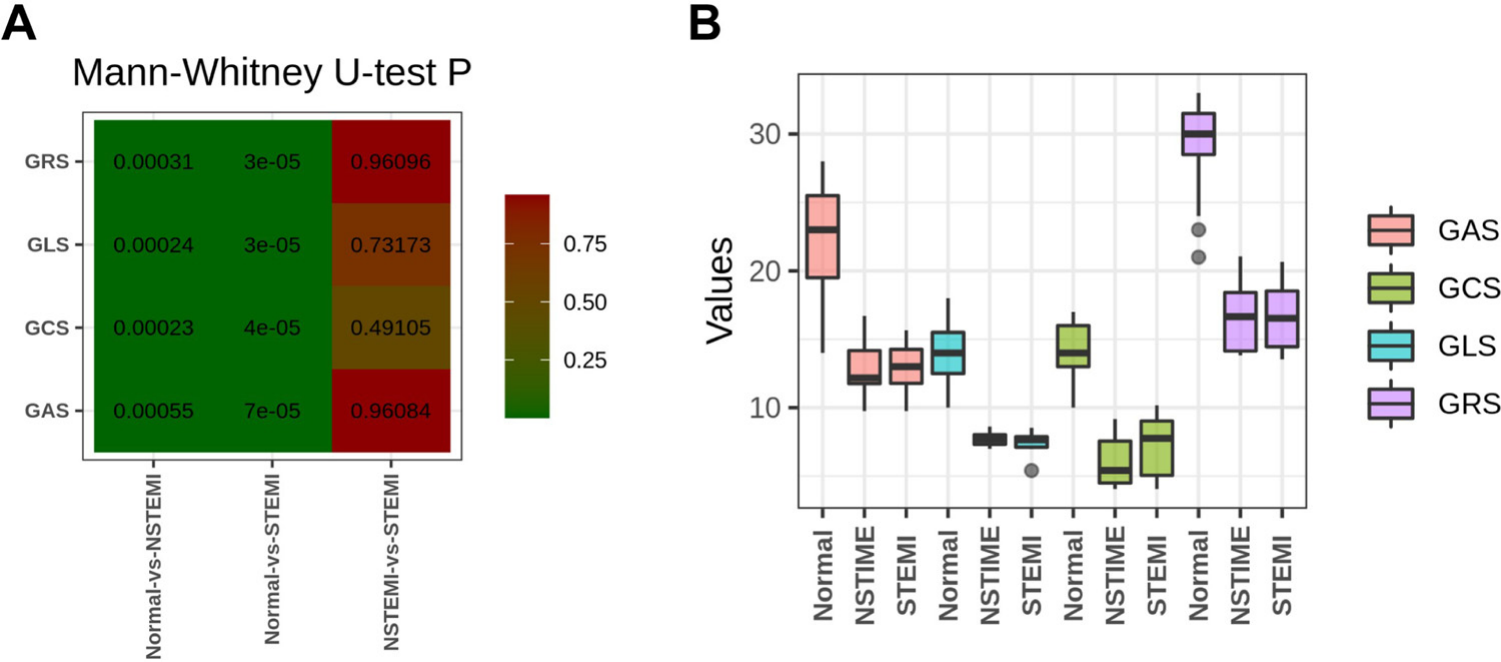
Discrimination effect of RT3D-STE strain index on STEMI group, NSTEMI group and normal group. (A) Four indicators distinguish the three groups. (B) Pairwise comparison of Mann-Whitney *U*-test results between each group in (A).

The clinical data of the three groups are shown in Table 1. The myocardial enzymes and myocardial binomial of the STEMI and NSTEMI groups are significantly higher than those of the normal group, while the STEMI group was higher than the NSTEMI group. RT3D-STE's strain parameters were GLS, GAS, GRS, and GCS, each index can significantly distinguish MI patients from healthy people, but none of them distinguished between NSTEMI and STEMI (Fig. 1).

### Identification of exosomes extracted from plasma

**Fig. 2:**
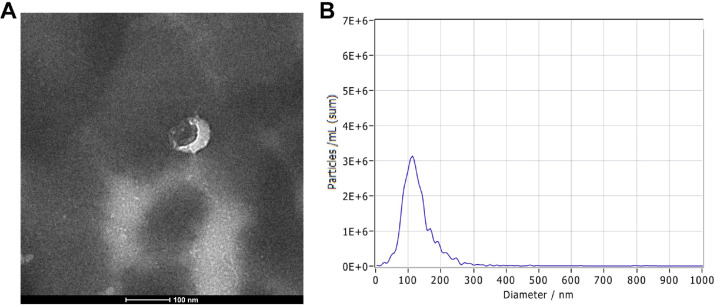
Identification of plasma exosomes. (A) Exosomes were photographed by transmission electron microscopy. (B) Concentration and diameter of isolated exosomes as detected by Nanoparticle Tracking Analysis (NTA).

The purification of plasma exosomes was observed by transmission electron microscopy. The purification products of exosomes contained a vesicle-like structure, and the expected size was 50‒200 nm, which is consistent with the morphology and size of exosomes (Fig. 2A). Nanoparticle Tracking Analysis (NTA) was used to detect the concentration and diameter of exosomes (Fig. 2B). These results are consistent with previously reported characteristics of exosomes, which confirmed that the exosomes are successfully purified from plasma samples.

### Identification of differentially expressed miRNAs

**Fig. 3:**
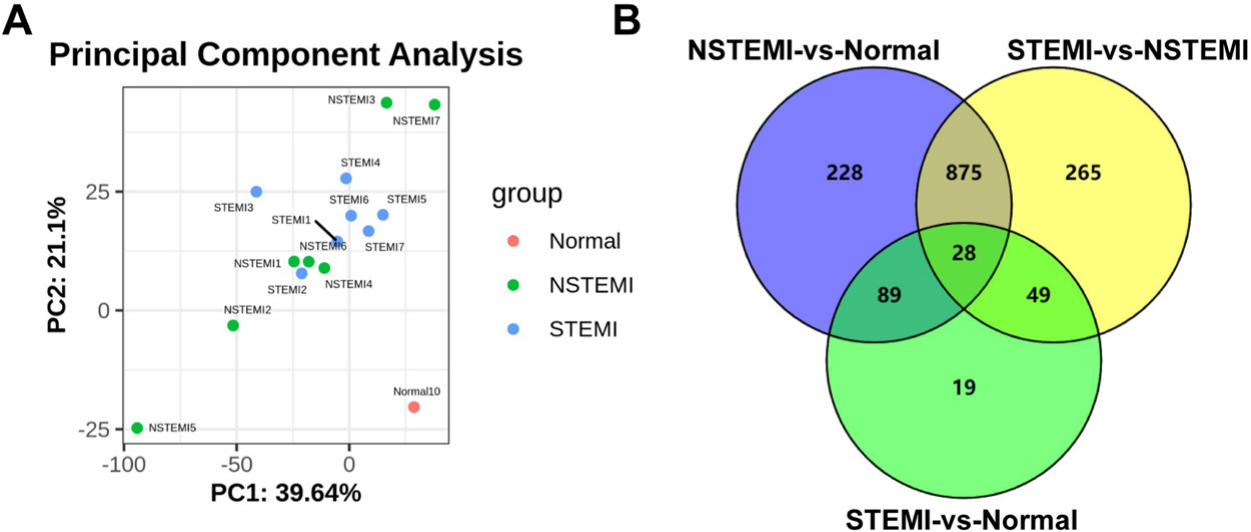
Identification of differentially expressed miRNAs. (A) Principal Components Analysis (PCA) of samples. (B)Venn diagrams of differently expressed miRNAs.

Libraries of 7 STEMI, 7 NSTEMI, and 10 normal plasmas were successfully sequenced. The differences between samples were verified by Principal Component Analysis (PCA), as shown in Fig. 3A. It showed that there were significant differences between the samples the authors collected. After bioinformatic analysis, a total of 2,571 miRNAs were annotated, which were considered to be known miRNAs. The Venn diagram was used to compare the differentially expressed genes of the three pairwise comparisons, and there was 28 identical differentially expressed miRNAs in each pairwise comparison (Fig. 3B). Compared with normal plasma, 24 miRNAs were upregulated, and 4 miRNAs were downregulated both in plasma exosomes from STEMI and NSTEMI, respectively. Among these 28 miRNAs, there were 27 downregulated and 1 upregulated miRNA in plasma exosomes from STEMI compared with NSTEMI (Table 2).

**Table 2:** Differentially expressed miRNAs in each pairwise comparison.

miRNA	STEMI vs. normal		NSTEMI vs. normal		STEMI vs. NSTEMI	
	Log2 (fold change)	State	Log2 (fold change)	State	Log2 (fold change)	State
miR-125a-5p	1.94	Up	4.02	Up	-2.42	Down
miR-193a-5p	3.35	Up	6.44	Up	-3.47	Down
miR-152-5p	-8.87	Down	-6.57	Down	-2.45	Down
miR-100-5p	2.55	Up	5.99	Up	-3.79	Down
miR-4286	2.01	Up	5.00	Up	-3.22	Down
miR-99a-5p	2.02	Up	5.98	Up	-4.36	Down
miR-365a-3p	2.41	Up	5.35	Up	-3.30	Down
miR-22-3p	2.36	Up	6.05	Up	-4.08	Down
miR-3168	6.82	Up	3.17	Up	3.49	Up
miR-3692-5p	1.53	Up	4.59	Up	-3.27	Down
miR-193b-3p	2.94	Up	4.95	Up	-2.37	Down
miR-1260b	2.14	Up	5.59	Up	-3.68	Down
miR-654-3p	1.16	Up	3.21	Up	-2.25	Down
miR-193b-5p	2.74	Up	5.58	Up	-3.21	Down
miR-885-5p	2.74	Up	5.76	Up	-3.33	Down
miR-122-5p	5.08	Up	8.52	Up	-3.81	Down
miR-29a-3p	1.07	Up	3.92	Up	-3.24	Down
miR-5579-5p	-6.77	Down	-1.83	Down	-4.38	Down
miR-361-5p	1.21	Up	4.06	Up	-3.17	Down
miR-574-3p	1.84	Up	4.24	Up	-2.68	Down
miR-6763-3p	3.72	Up	6.43	Up	-2.90	Down
miR-1246	2.34	Up	4.85	Up	-2.88	Down
miR-133a-3p	1.87	Up	4.09	Up	-2.59	Down
let-7d-3p	2.13	Up	4.23	Up	-2.39	Down
miR-3681-5p	-4.09	Down	-1.69	Down	-2.69	Down
miR-4520-2-3p	-4.32	Down	-2.19	Down	-2.28	Down
miR-320b	1.51	Up	3.70	Up	-2.51	Down
miR-345-5p	1.35	Up	4.49	Up	-3.53	Down

### The correlation analysis between strain parameters of RT3D-STE and miRNAs

A total of 28 differentially expressed miRNAs were selected for correlation analysis with Strain parameters of RT3D-STE. The absolute value of Pearson correlation > 0.6, p-value < 0.05, is considered a strong correlation. There are 10 miRNAs (miR-152-5p, miR-3681-5p, miR-193a-5p, miR-193b-5p miR-345-5p, miR-125a-5p, miR-365a-3p, miR-4520-2-3p, miR-193b-3p and miR-5579-5p) which had strong correlation with GAS, GLS, GCS and GRS, and these miRNAs are all down-regulated in STEMI compared with NSTEMI (Table 3). Therefore, these miRNAs can be used as important markers for early clinical diagnosis of STEMI and NSTEMI.

**Table 3:** Correlation analysis between clinical traits and miRNA.

Clinical traits	miRNA	Pearson correlation	p-value	STEMI vs. normal	NSTEMI vs. normal	STEMI vs. NSTEMI
GAS	miR-193a-5p	0.605654207	0.002193818	Up	Up	Down
GAS	miR-152-5p	-0.865219779	9.91E-08	Down	Down	Down
GAS	miR-365a-3p	0.684190251	0.000317771	Up	Up	Down
GAS	miR-193b-3p	0.623437872	0.001481382	Up	Up	Down
GAS	miR-3681-5p	-0.843457274	4.32E-07	Down	Down	Down
GAS	miR-4520-2-3p	-0.76151602	2.43E-05	Down	Down	Down
GAS	miR-345-5p	0.628820483	0.001309249	Up	Up	Down
GLS	miR-125a-5p	0.662665432	0.000569943	Up	Up	Down
GLS	miR-152-5p	-0.841408197	4.90E-07	Down	Down	Down
GLS	miR-193b-3p	0.641732861	0.00096437	Up	Up	Down
GLS	miR-3681-5p	-0.790501784	7.17E-06	Down	Down	Down
GLS	hsa-miR-4520-2-3p	-0.636664236	0.001089103	Down	Down	Down
GLS	miR-345-5p	0.709916244	0.000147959	Up	Up	Down
GCS	miR-193a-5p	0.626242438	0.001389433	Up	Up	Down
GCS	miR-152-5p	-0.795081017	5.81E-06	Down	Down	Down
GCS	miR-365a-3p	0.685366976	0.000307358	Up	Up	Down
GCS	miR-193b-3p	0.685069136	0.000309966	Up	Up	Down
GCS	miR-193b-5p	0.623812538	0.001468806	Up	Up	Down
GCS	miR-3681-5p	-0.756184148	2.99E-05	Down	Down	Down
GCS	miR-4520-2-3p	-0.636572173	0.00109149	Down	Down	Down
GCS	miR-345-5p	0.690394623	0.000266123	Up	Up	Down
GRS	miR-125a-5p	-0.667331646	0.000504119	Up	Up	Down
GRS	miR-193a-5p	-0.66530648	0.000531834	Up	Up	Down
GRS	miR-152-5p	0.873020075	5.49E-08	Down	Down	Down
GRS	miR-365a-3p	-0.763583867	2.24E-05	Up	Up	Down
GRS	miR-193b-3p	-0.68558743	0.000305441	Up	Up	Down
GRS	miR-5579-5p	0.619218121	0.001629477	Down	Down	Down
GRS	miR-3681-5p	0.827378244	1.12E-06	Down	Down	Down
GRS	miR-4520-2-3p	0.743215454	4.84E-05	Down	Down	Down
GRS	miR-345-5p	-0.728936463	7.97E-05	Up	Up	Down

### The prediction of target genes of differentially expressed miRNAs and functional enrichment analysis

**Fig. 4:**
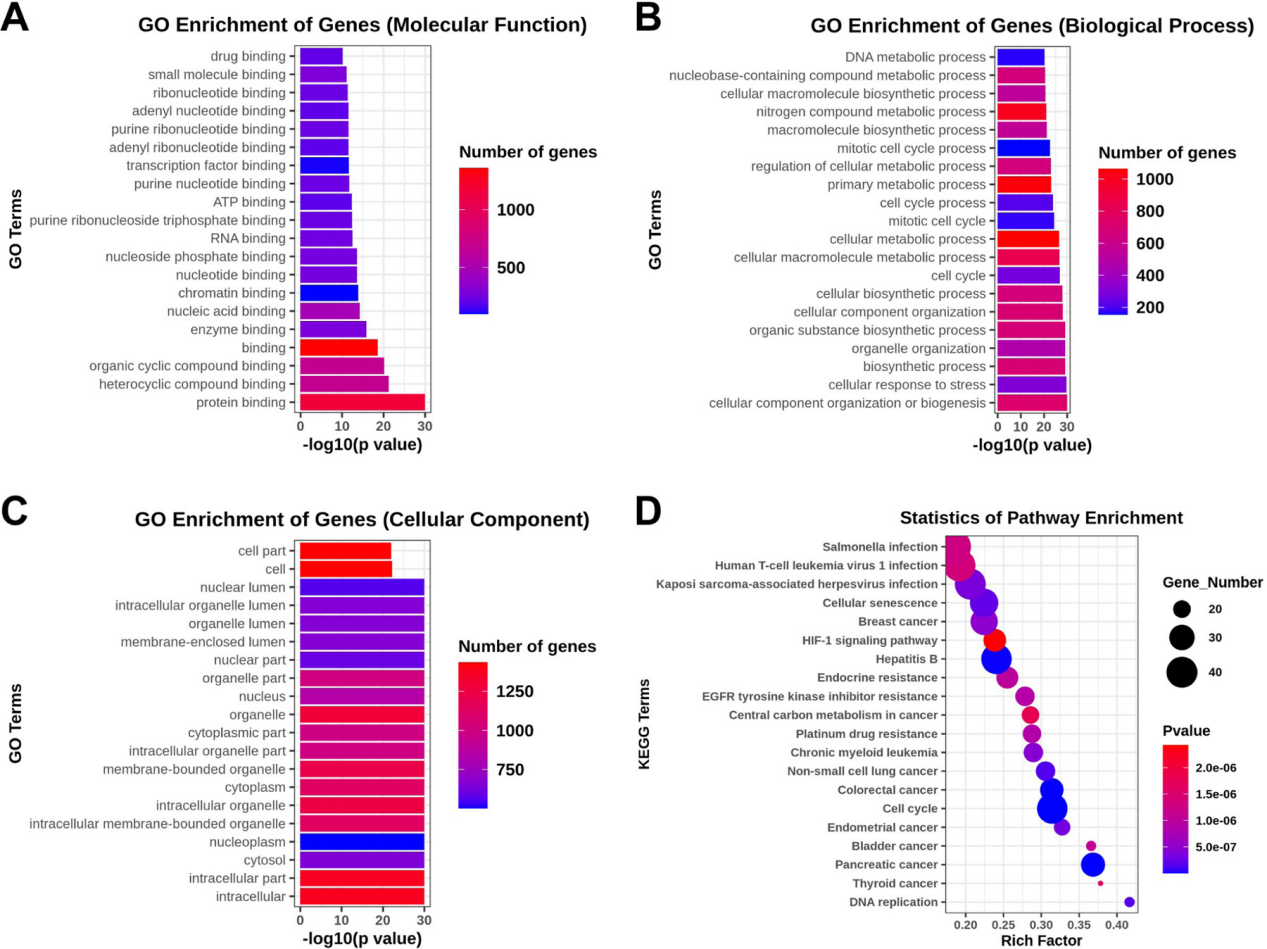
Top 20 functional GO terms and KEGG pathways of the differentially expressed miRNA target genes shown according to the p-value. (A) Molecular Function terms, (B) Biological Process terms, (C) Cellular Component terms, (D) Top 20 KEGG pathways.

The GO analysis, including the biological process, cellular component, and molecular function, was performed to investigate the function and annotation of the target genes. The 20 terms that were most significantly enriched in these three domains are presented in Fig. 4A‒C. The differentially expressed genes were closely correlated with the following molecular functions: protein binding, heterocycle compound binding, organic compound binding, binding, and enzyme binding. The involved primary biological processes were cellular component organization or biogenesis, cellular response to stress, biosynthetic process, organelle organization, and organic substance biosynthetic process. The cellular components most strongly associated with the differentially expressed genes were intracellular, intracellular part, cytosol, nucleoplasm, and intracellular membrane-bounded organelle. The investigators drew scatter plots to intuitively illustrate the KEGG analysis results. The 20 pathways that exhibited a smaller p-value are shown in Fig. 4D. The analysis indicated that the signaling pathways with significant correlations included the HIF-1 signaling pathway.

### RT-qPCR validation of the differentially expressed miRNAs

**Fig. 5:**
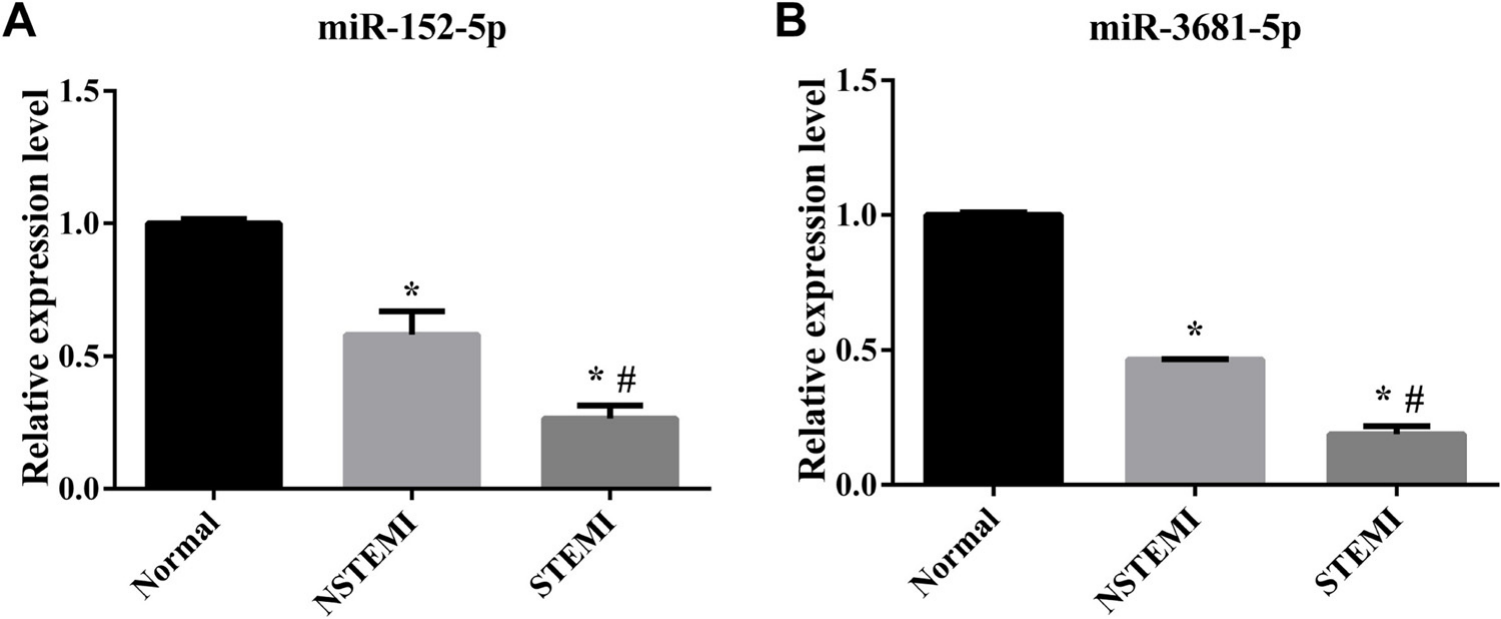
The sequencing results were verified by RT-qPCR. (A) The relative Expression level of miR-152-5p in each group; (B) Relative Expression level of miR-3681-5p in each group (n=3 biological replicates for each assay, mean ± SEM, * compared with Normal p<0.05, **^#^** compared with NSTEMI p<0.05 one-way AVOVA).

The authors employed RT-qPCR to verify the expression levels of 2 randomly selected differentially expressed miRNAs. RT-qPCR results exhibited the same trend of expression changes as the high-throughput sequencing analysis. Specifically, as shown in Fig. 5, the expression levels of miR-152-5p and miR-3681-5p were decreased in each pairwise comparison.

## Discussion

Diagnosing STEMI and NSTEMI immediately after symptoms appear may reduce mortality and improve patient prognosis. RT3D-STE can track the movement of myocardial spots in three-dimensional space and simultaneously measure the longitudinal, radial, circumferential, and regional strains of the left ventricle, which can quantitatively evaluate the range of AMI infarct area and the overall and local myocardial functions of the left ventricle. It may become a visual quantitative tool for the clinical diagnosis of AMI.^12^^,^^16^ In the present study, each index (GLS, GAS, GRS, and GCS) can significantly distinguish MI patients from healthy people, but none of them can distinguish between NSTEMI and STEMI. Exosomes are considered intercellular communicators, which can play an important role in the diagnosis and therapy of acute myocardial infarctions. miRNAs have been demonstrated to play important roles in cell biological and pathological processes, including proliferation, differentiation, and apoptosis.^17^^,^^18^ A large number of studies have shown that miRNAs are implicated in the pathogenesis of various cardiovascular diseases or disorders. Moreover, accumulating evidence has indicated that exosome miRNAs have the potential to serve as alternative biomarkers for the diagnosis of AMI.^19^, ^20^, ^21^ This is the first study to analyze the correlation between differentially expressed exosome miRNAs and Strain parameters of RT3D-STE in STEMI patients and NSTEMI patients.

In this study, the authors have identified 10 differentially expressed plasma exosome miRNAs in STEMI and NSTEM, which had a strong correlation with GAS, GLS, GCS, and GRS. These miRNAs may be biomarkers for the diagnosis of STEMI and NSTEMI. Several miRNAs identified in this analysis have also been reported to play an important role in cardiovascular disease in previous studies. Wong et al.^22^ found that miR-193b-5p, miR-125a-5p, and miR-193b-3p could differentiate heart failure from non-heart failure, and they may complement the diagnostic value of BNP/NT-proBNP. Mansueto et al.^23^ suggested that miR-125a-5p combination with other miRNAs (such as miR-520d-5p and miR-190a) may aid in discriminating different phenotypes of heart failure ranging from preserved to reduced ejection fraction. Huan et al.^24^ observed that miR-365a-3p was differentially expressed in coronary heart disease cases than in controls and associated with an mRNA co-expression module that was causally linked to coronary heart disease. Yan et al.^25^ demonstrated that long noncoding RNA NEAT1 sponges miR‑125a‑5p to suppress cardiomyocyte apoptosis via BCL2L12. AMI is often accompanied by inflammation and endothelial cell damage, and some of these miRNAs have been reported to be associated with these disorders.^26^^,^^27^ miR-345-5p^28^ and miR-365a-3p^29^ Can inhibit myocardial cell inflammation. Bai et al.^30^ found that knockdown of miR-193b-3p suppressed the oxidized low-density lipoprotein-induced human umbilical vein endothelial cells injury. Cao et al.^31^ suggested that circulating exosomes could shuttle between cells through dynamin and deliver miR-193a-5p to protect endothelial cells from oxidative stress damage via activin a receptor type I in AMI convalescence. These results suggest that miR-152-5p, miR-3681-5p, miR-193a-5p, miR-193b-5p miR-345-5p, miR-125a-5p, miR-365a-3p, miR-4520-2-3p, hsa-miR-193b-3p and hsa-miR-5579-5p combined with RT3D-STE may have important value in early diagnosis of STEMI and NSTEMI.

Among these 10 miRNAs, miR-152-5p and miR-3681-5p had a strong correlation with the strain parameters of RT3D-STE. These two miRNAs have not been reported in heart-related diseases before. Wang et al.^32^ found that melatonin treatment of focal cerebral ischemia model rats reduced the expression of plasma exosomal miR-152-5p, and could reduce the inflammatory response after stroke through the TLR4/NF-κB pathway. miR-152-5p can inhibit the secretion of pro-inflammatory factors in microglial BV2 cells.^33^ The expression of miR-3681-5p is upregulated in celiac disease patients, which may be related to immunity and intestinal inflammation.^34^^,^^35^ Therefore, miR-152-5p and miR-3681-5p may play an important role in inflammatory response elicited by myocardial infarction and may function as potential biomarkers for ST-segment elevation myocardial infarction.

The GO, through the test function analysis, revealed that the AMI related to differentially expressed miRNAs are mainly involved in biological processes, which include cellular metabolism and cell cycle. The KEGG pathway analysis revealed that HIF-1 signaling pathway plays an important role in the occurrence and development of STEMI and NSTEMI. HIF-1 signaling pathway is a stress signaling pathway in hypoxia, which can protect the myocardium by regulating the function of HIF-1α.^36^^,^^37^ Under hypoxia, HIF-1α accumulates, promotes the division and proliferation of vascular endothelial cells, induces angiogenesis and regeneration, inhibits inflammation after vascular endothelial cell injury, and maintains the function of cardiomyocytes.^38^, ^39^, ^40^ Myocardial hypoxia-ischemia is the main mechanism of myocardial infarction, and the degree of ischemia is different between STEMI and NSTEMI. HIF-1 signaling pathway is an important signaling pathway in STEMI and NSTEMI that needs to study further.

Therefore, to some extent, the present results show that RT3D-STE combined with miRNAs detection may have great academic research value and the clinical application potential in studying the role of heart-specific miRNAs in the early diagnosis and prognostic evaluation of STEMI and NSTEMI, exploring their value in assisting in early diagnosis of AMI, quantitatively evaluating the risk of its condition, discussing the evaluation indicators for risk stratification, and coordinating the current clinical applications of ACS risk assessment.

## Conclusion

RT3D-STE has advantages of convenience, quickness, and real-time results, but it cannot distinguish STEMI from NSTEMI patients. There are many types of exosomal miRNAs, which can reflect the physiological state of living cells, and the 10 exosomal miRNAs (miR-152-5p, miR-3681-5p, miR-193a-5p, miR-193b-5p miR-345-5p, miR-125a-5p, miR-365a-3p, miR-4520-2-3p, hsa-miR-193b-3p, and hsa-miR-5579-5p) identified in this study can accurately distinguish STEMI from NSTEMI, which are stronger than RT3D-STE, and are expected to provide warning earlier than RT3D-STE. However, exosomal miRNAs need to go through multiple steps such as exosomal isolation, RNA isolation, and RT-qPCR. The operation is precise and cumbersome and requires high operators, so the cost is high, and the results cannot be obtained in real time. Therefore, RT3D-STE and exosomal miRNAs can be used as a hierarchical diagnosis system. When the RT3D-STE check is abnormal, the exosomal miRNAs can be detected to obtain more detailed and accurate diagnosis results. This is a combination of diagnostic accuracy and a comprehensive plan of cost. miR-152-5p and miR-3681-5p may play an important role in the inflammatory response induced by myocardial infarction and may serve as potential biomarkers for ST-segment elevation myocardial infarction. Next, cell experiments should be carried out to further verify the roles of miR-152-5p and miR-3681-5p in inflammation, hypercoagulability, and endothelial injury caused by AMI.

## Institutional review board statement

The present study was reviewed and approved by the Research Ethics Committee of Shenzhen Longhua People's Hospital (n° KY20200801).

## Ethical declaration found

The present study was approved by the Research Ethics Committee of Shenzhen Longhua People's Hospital (n° KY20200801). All ethical procedures conformed with the principles of the 1964 Declaration of Helsinki and its latest 2008 amendments.

## Data statement

All data generated or analyzed during this study are included in this article.

## Patient consent for publication

All the patients agreed to publish.

## CRediT authorship contribution statement

**Xiaozhu Chen:** Conceptualization, Methodology, Writing – original draft. **Fengrong Huang:** Methodology, Formal analysis, Writing – original draft. **Yunhong Liu:** Investigation, Visualization. **Shujun Liu:** Software, Formal analysis. **Gangwen Tan:** Investigation, Visualization.

## Conflicts of interest

The authors declare no conflicts of interest.

## Appendix Supplementary materials

Image, application 1
