# Supplementary material for: Exosomal miR-152-5p and miR-3681-5p function as potential biomarkers for ST-segment elevation myocardial infarction
Source: Clinics (Sao Paulo). 2022 Jun 22;77:100038. doi: 10.1016/j.clinsp.2022.100038 (PMC9243048; doi:10.1016/j.clinsp.2022.100038)
Supplement: Supplementary file 1 [file mmc1.pdf]

Supporting Table 1 Primer sequences of identified genes.

**miRNA**	Primer sequence (**5`–3`**)
**H-U6**	CTCGCTTCGGCAGCACAT
**miR-152-5p**	AGGTTCTGTGATACACTCCGACT
**miR-3681-5p**	TAGTGGATGATGCACTCTGTGC
